# Effect of *Siraitia grosvenorii* Polysaccharide on Glucose and Lipid of Diabetic Rabbits Induced by Feeding High Fat/High Sucrose Chow

**DOI:** 10.1155/2007/67435

**Published:** 2007-12-09

**Authors:** Guo-Ping Lin, Tao Jiang, Xiao-Bo Hu, Xin-Hui Qiao, Qin-Hui Tuo

**Affiliations:** School of Life Science and Technology, University of South China, Hengyang 421001, Hunan, China

## Abstract

The *Siraitia grosvenorii* polysaccharide (SGP) from the *Siraitia grosvenorii* (Swingle) was isolated and purified. The therapeutic effects of SGP on diabetic rabbits induced by feeding high fat/high sucrose chow were studied. After administration of SGP for 4 weeks, the fasting blood glucose (FBG), plasma insulin levels (INS), plasma total cholesterol (TC), triglyceride (TG), and HDL-C were assayed. The results showed that administration of SGP can significantly decrease plasma total cholesterol, triglyceride, and glucose levels; and increase HDL-C levels after 4 weeks of treatment. The antihyperglycaemic effect of SGP at dose of 100 mg·kg^−1^ bw was the most significant in three dosage groups. Furthermore, SGP could restore the blood lipid levels of diabetic rabbits (P<.05). These data indicate that SGP not only ameliorates the lipid disorder, but also lowers plasma glucose levels. So SGP have obvious glucose-lowering effect on hyperglycaemic rabbits induced by feeding high fat/high sucrose chow, its mechanism may be related to amelioration of lipid metabolism and restoring the blood lipid levels of hyperglycaemic rabbits.

## 1. INTRODUCTION

Diabetes is a common endocrine-metabolic disease with rising incidence in recent years. It is the third most life-threatening disease whose mortality is right after cancer and cardiovascular disease. Research and development of drugs against diabetes and its complications have been getting more and more attentions. Many types of polysaccharides, isolated from herbal medicine, have been proved to have effects of lowering the blood glucose and lipids level in animal models of diabetes [1–4]. Progresses in research of these herbal medicines may open up new and important ways in treating diabetes.

Mangosteen is a medicinal plant unique in China, mainly in the southern Guangxi and Hunan, with features of tasted sweet, being cool, and nontoxic. In clinical practice, it can heal cough, relieve fever, and promote digestion/excretion. It has been widely used in the treatment of laryngitis, bronchitis, and gastrointestinal diseases in traditional Chinese medicine. The previous study about Mangosteen has been confined to its sweet glycoside which account for about only 2% of its dry weight [1–3], without much being mentioned about its polysaccharide components purification and biological activity. This research was focused on purification of polysaccharide components, its regulatory role on high-fat- high-sugar-induced diabetes in New Zealand's rabbits.

## 2. MATERIALS AND METHODS

### 2.1. Materials

*Siraitia grosvenorii* was obtained from the Hengyang Medicine Company (Hunan, China). Sucrose was obtained from Liuzhou Sugar Company (Guangxi, China). Lard was obtained from Hengyang Meat Product Company (Hunan, China). Sephadex G-200 column and DEAE-cellulose column were from Whatman (Beijing, China). All other chemicals used were high-grade commercially available products.

### 2.2. Isolation and purification of the *Siraitia grosvenorii* polysaccharide (SGP)

The polysaccharides were extracted from *Siraitia grosvenorii* with boiling water or enzyme digestion with pancreatin. In brief, dry fruit of the plant (100 g) was fragmented and boiled in water for 5 hours. Filtrate was collected after it was cooled to room temperature; or pancreatin was added to the 100 g fragmented dry fruit and stirred at 40°C for 4 hours. Filtrate was collected after 1-hour boiling to inactivate the pancreatin and cooling to room temperature. The crude polysaccharides were obtained by 95% ethanol precipitation overnight followed by washing with ethanol/acetone and vacuum drying. DEAE chromatography was performed after the precipitate was dissolved with H_2_O. Elute peaks, monitored using Sulfate-Phenol method, were combined and applied to Sephadex G-200. Peaks were eluted with H_2_O or normal saline and combined, followed by 95% ethanol precipitation, washing with acetone/ether, and vacuum drying. The *Siraitia grosvenorii* polysaccharide (SGP) was obtained and the yield was 1.2% (extracted with boiling water) and 2.3% (extracted enzyme digestion with pancreatin). The total carbohydrate amount was 46.8% and 42.6%, respectively, using Sulfate-Phenol measurement. UV scanning spectrum shows that there are no absorption peaks of protein and nuclear acid at 280 nm and 260 nm.

### 2.3. Animals experiments

Male New Zealand white rabbits weighing approximately 2 kg were obtained from the Shen Wan Ranch (Shanghai, China). The animals were maintained at a 12-hour light-dark cycle and a constant temperature of 23±2°C. Thirty male New Zealand white rabbits were randomly assigned into five groups: group A: the normal control which received regular rabbit chow (Standard laboratory chow, Shen Wan Experimental Animal Ranch, Shanghai, China) for 8 weeks (Normal group); group B: the control which was fed high fat/high sucrose chow (incorporating 10% lard and 37% sucrose into the standard laboratory chow [4, 5], prepared in our institute) for 8 weeks (Control group). group C-E: the treated which was fed high fat/high sucrose chow (incorporating 10% lard and 37% sucrose into the standard laboratory chow) for the first 4 weeks. From beginning of week 5, SGP at dose of 50, 100, 200 mg·kg^-1^ bw were supplemented, respectively, into the high fat/high sucrose chow for the remaining 4 weeks (SGP group: group C: at dose of 50 mg·kg^-1^ bw SGP group; group D: 100 mg·kg^-1^ bw SGP group; group E: 200 mg·kg^-1^ bw SGP group); All 5 groups were fed an amount of 35 g/kg/day (g per kg body weight and day). The animals were fed at 9 am and given free access to tap water. Food consumption was measured daily. Blood samples for glucose, lipid, and insulin measurements were collected from auricular veins after fasting overnight.

All animal experiments were approved by the local animal ethics committee of University of South China.

### 2.4. Analytical methods

Plasma total cholesterol (TC-test kit), HDL-C (HDL-C-test kit), triglycerides (TG-test kit), and glucose (glucose oxidase-peroxidase method) were determined by commercially available enzymatic methods (Shanghai Rongsheng Biotech Inc, Shanghai, China). Insulin was determined by conventional radioimmunoassay, with the use of Insulin radioimmunology kit (Beijing Institute of Atomic Research, Beijing, China).

### 2.5. Statistical analysis

Values are reported as means ±SD. Comparisons between groups were analyzed for statistical significance using the one-way analysis of variance, followed by the Dunnett's test multiple comparisons. P values less than .05 were considered significant.

## 3. RESULTS AND DISCUSSION

### 3.1. Effects of SGP on fasting blood glucose and insulin levels

Fasting blood glucose level in rabbits from group B (the Control group) was the highest among 5 groups by the 8 weeks of high fat/high sucrose feeding. However, the increase of glucose level was inhibited by supplementing SGP (SGP group). SGP at dose of 100, 200 mg·kg^-1^ bw significantly lowered the fasting blood glucose and the antihyperglycaemic effect of SGP at dose of 100 mg·kg^-1^ bw was the most significant in three dosage groups (see Table 1).

There were no differences in plasma insulin levels among the normal group, the control group, and the SGP group during the experimental period (see Table 1).

### 3.2. Effects of SGP on plasma lipid levels

Plasma total cholesterol levels in the control group were higher than those in the normal group. Plasma total cholesterol levels in the SGP group were lower than those in the control group. Plasma triglyceride levels in the control group were higher than those in the normal group. Plasma triglyceride levels in the SGP group were lower than those in the control group. Plasma HDL-C levels in the control group were lower than those in the normal group. Plasma HDL-C levels in the SGP group were higher than those in the control group (see Table 2).

Feeding high fat/high sucrose diets elevated plasma total cholesterol, triglyceride levels, and decreased HDL-C levels. However, these changes were inhibited by supplementing SGP into the high fat/high sucrose diets, and the effect of SGP at dose of 100 mg·kg^-1^ bw was the most significant within the three dosage group.

The results are shown in Tables 1 and 2. High fat/high sucrose increased plasma total cholesterol, triglyceride, and glucose levels; and decreased HDL-C levels resulting in atherosclerosis in the aorta. Administration of SGP to the rabbits resulted in decreased plasma total cholesterol, triglyceride, and glucose levels; and increased HDL-C levels after 4 weeks of treatment. There data indicate that SGP not only ameliorates the lipid disorder, but also lowers plasma glucose levels.

### 3.3. Discussion

Lipid disorder in diabetic subjects is a high risk factor for cardiovascular disease. Diabetes is a glucose disorder and is usually accompanied with a lipid disorder. This may suggest that the reduction of insulin sensitivity causes both glucose disorder and lipid disorder. Accordingly, this relationship may indicate that amelioration of the glucose disorder induces amelioration of the lipid disorder, and conversely, amelioration of the lipid disorder induces amelioration of the glucose disorder.

In this study, we used high fat/high sucrose feeding-induced type 2 diabetes rabbit model to determine the effects of SGP on atherosclerosis. In general, cholesterol-fed rabbits have been a widely used model for experimental atherosclerosis research [6]. Our rabbit model showed mild hypercholesterolemia and hypertriglyceridemia (control group, total cholesterol level: 2.76 mmol·L^-1^, triglyceride level: 2.46 mmol·L^-1^). This model also showed increased plasma glucose levels (control group: 7.36±0.76 mmol·L^-1^ versuss normal group: 4.31±0.58). Therefore, the characteristics of this model are mild type 2 diabetes with high plasma glucose levels. Administration of SGP can significantly decrease plasma total cholesterol, triglyceride, and glucose levels and elevate HDL-C levels after 4 weeks of treatment in this rabbit model. Furthermore, SGP could restore the blood lipid levels of diabetic rabbits (*P* < .05). This means that SGP has a therapeutic effect by inhibiting the increase in of plasma glucose in this rabbit model. However, this effect did not cause the plasma glucose levels to drop to levels lower than normal (normal group: 4.31±0.58 mmol·L^-1^ versus SGP group: 4.52±0.37 mmol·L^-1^).

These data indicate that SGP not only ameliorates the lipid disorder, but also lowers plasma glucose levels. So SGP have obvious glucose-lowering effect on hyperglycaemic rabbits induced by feeding high fat/high sucrose chow, its mechanism may be related to amelioration of lipid metabolism and restoring the blood lipid levels of hyperglycaemic rabbits.

However, there were no differences in plasma insulin levels among the normal group, the control group, and the SGP group during the experimental period. We did not observe any significant effect of SGP on fasting insulin, so the decrease of fasting glucose might be due to improved insulin sensitivity through the actions of SGP on plasma triglycerides as shown in this study.

To our knowledge, this is the first report in the world about purification of polysaccharides components from *Siraitia grosvenorii* by enzyme digestion/ethanol precipitation and DEAE/Sephadex G-200 chromatography. The polysaccharides components SGP belong to acidic hetero-polysaccharides, which composed of glucose, maltose, galactose, arabinose, and so forth. They are potential polysaccharide drugs which need further research about their composition and pharmacological effect.

In summary, SGP significantly decreased plasma total cholesterol, triglyceride, and glucose levels and elevated HDL-C levels in rabbits with high fat/high sucrose-feeding-induced mild diabetes type 2. Therefore, SGP is potentially beneficial for the treatment of hyperglycaemia and lipid disorder which are commonly associated with diabetes.

## Figures and Tables

**Table 1:** Effects of SGP on fasting glucose and insulin levels after high fat/high sucrose feeding in New Zealand white rabbits (n=6 per group). Data are expressed as means ±SD.*P < .05 and **P < .01 versus control group.

Group	Dose	fasting blood glucose	plasma insulin levels
	mg·kg^-1^ bw	/mmol·L^-1^	/*μ*U·ml^-1^
A (normal group)	—	4.31±0.58**	14.4±9.8
B (control group)	—	7.36±0.76	13.8±12.5
C (SGP group)	50	6.18±0.43*	17.6±8.7
D (SGP group)	100	4.52±0.37**	14.9±7.9
E (SGP group)	200	4.68±0.81**	17.0±10.5

**Table 2:** Effects of SGP on plasma lipid levels after high fat/high sucrose feeding in New Zealand White rabbits (n=6 per group). Data are expressed as means ±SD. *P < .05 and **P < .01 versus Control group, PΔ<0.1 versus SGP 100 group.

Group	Dose	TC	TG	HDL-C
	mg·kg^-1^ bw	mmol·L^-1^	mmol·L^-1^	mmol·L^-1^
A (Normal group)	—	$1.75\pm 0.26^{**\Delta }$	$0.94\pm 0.16^{**\Delta }$	$1.62\pm 0.21^{**\Delta }$
B (Control group)	—	$2.76\pm 0.43^{\Delta }$	$2.46\pm 0.53^{\Delta }$	$0.89\pm 0.15^{\Delta }$
C (SGP group)	50	$2.54\pm 0.31^{\Delta }$	$2.04\pm 0.36^{\Delta }$	$1.10\pm 0.18^{\Delta }$
D (SGP group)	100	$2.03\pm 0.23^{*}$	$1.56\pm 0.27^{**}$	$1.47\pm 0.14^{**}$
E (SGP group)	200	$2.01\pm 0.25^{*}$	$1.57\pm 0.28^{**}$	$1.50\pm 0.15^{**}$
